# Primary non Hodgkin's lymphoma of the lacrimal sac

**DOI:** 10.1186/1477-7819-5-127

**Published:** 2007-11-06

**Authors:** Ramachandran Venkitaraman, Mathew K George

**Affiliations:** 1 Division of Clinical Oncology, Royal Marsden Hospital, Sutton, Surrey, SM2 5PT, UK; 2 Division of Medical Oncology, Royal Darwin Hospital, Casuarina, Australia

## Abstract

**Background:**

Primary Non Hodgkin's Lymphoma (NHL) of the lacrimal sac is rare.

**Methods:**

The clinical features of a 78 year old female who presented with epiphora and swelling of the left lacrimal sac are described.

**Results:**

Computerised tomography showed a mass involving the left lacrimal sac. Histopathological examination revealed a diffuse large B cell NHL. Immunohistological examination demonstrated B cell origin. Chemotherapy could not be administered due to co morbid conditions. The patient was treated with radiotherapy to a dose of 45 Gy in 25 fractions. Patient is disease free and on follow up after 36 months.

**Conclusion:**

Primary radiotherapy is a treatment option with curative potential for localized NHL of the lacrimal sac and may be considered in patients who cannot tolerate appropriate chemotherapy.

## Background

Tumors involving the nasolacrimal drainage system are rare and more than 90% of these tumours are of epithelial origin [1]. Majority of lymphomas involving the lacrimal sac are secondary to systemic lymphoreticular malignancy [1]. Primary non Hodgkin's lymphoma (NHL) of the lacrimal sac is a rare neoplasm [2-6]. Therefore, the clinicopathological features and optimal management of primary lacrimal sac NHL are not well characterized.

In this report, we present the clinical features of a patient with primary NHL of the lacrimal sac treated successfully with radiotherapy.

## Case presentation

**Figure 1:**
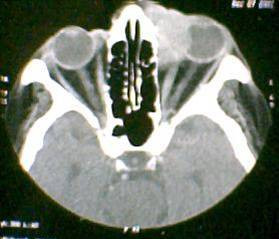
Computerised Tomograph of the orbit showing the lacrimal sac tumour with contrast enhancement.

A 78 year old female patient presented with epiphora of the left eye of 6 months duration and a painless swelling of the left lacrimal sac area of 2 months duration. Physical examination showed a firm, non-tender mass measuring 3 × 4 cm in the right lacrimal fossa. Irrigation test revealed complete obstruction of the lacrimal drainage system. Rest of the eye and adnexae were normal. Computerised tomography (CT) scan of the orbit showed a heterogeneous soft tissue mass arising from the lacrimal sac, infiltrating and pushing the globe laterally and forward, causing exophthalmos with no bony erosion (figure 1). There was homogenous and intense enhancement with contrast.

An incision biopsy was performed from the mass and pathological examination of revealed diffuse large cell lymphoma. Immunohistochemistry demonstrated malignant clonal cells of B cell origin (CD20 positive), lacking T cell antigens. Physical examination, biochemical tests, CT scan of the chest and abdomen, and bone marrow biopsy did not show evidence of lymphomatous involvement elsewhere. The patient had diffuse large B cell Non Hodgkin's lymphoma of the right lacrimal sac, stage IE by the Ann Arbor staging system and IPI score of 2 (low intermediate).

The patient was treated with radiotherapy to a dose of 45 Gy in 25 fractions over 5 weeks by three dimensional conformal lens sparing technique. Systemic chemotherapy was not proposed in view of the advanced age of the patient, poor performance status and concurrent co morbidities. The patient continues to be on follow up after 36 months with no evidence of local or systemic recurrence and preservation of visual acuity.

## Discussion

Primary lymphoma of the lacrimal sac is rare, with most reported cases representing secondary involvement from systemic lymphoproliferative malignancy [1]. Only 16 of 212 lacrimal sac tumors described by Flanagan and Stokes were lymphoreticular tumors [1]. In largest cumulative series of nonepithelial tumors of the lacrimal sac, Pe'er at al found that 8 of 35 cases were primary lymphoma [2].

The typical presentation of a lacrimal sac tumour is as a medial canthal swelling with nasolacrimal duct obstruction. The differentials for a lacrimal sac mass include nongranulomatous inflammation and granulomatous infections, dermoid cyst, mucocele, lipoma, lymphangioma and tumors of the lacrimal duct like adenocarcinoma, squamous cell carcinoma, adenoid cystic carcinoma, hemangiomas, sarcomas, melanoma, lymphoma and secondaries.

Nasolacrimal sac tumors may arise within the sac or from structures surrounding the sac or duct. In previous reports of primary NHL of the lacrimal sac, Jordan and Nerad [7] observed a lacrimal sac mass extending into the nasolacrimal duct, Saccogna *et al *[8] demonstrated CT findings of a large mass involving the nasolacrimal duct area and the adjacent paranasal sinuses and Erickson *et al *[9] treated several patients with lymphomatous infiltration of the nasolacrimal canal.

The vast majority of NHLs of the orbit and ocular adnexa are of B cell origin. T cell lesions are rare and usually are associated with disseminated cutaneous lymphomas, such as mycosis fungoides, Sézary syndrome, or adult T cell leukemia. Primary lacrimal sac lymphoma also appears to be commonly of B cell origin and the usual subtypes are either indolent types like MALT lymphoma or extranodal marginal B cell lymphoma or aggressive subtypes like diffuse large B cell lymphoma [7].

The treatment of lacrimal sac tumors of epithelial origin is mainly surgical. However, in cases of orbital lymphoma extensive surgery is to be avoided for adequate cosmesis and functional preservation of the eye [9-14].

Chemotherapy is considered the standard treatment for localized aggressive NHL with either CHOP based chemotherapy alone or limited cycles of chemotherapy followed by a lower dose of involved field radiotherapy after a good response [15]. Chemotherapy alone as primary treatment for Stage I orbital lymphomas has not been proven and is not advocated except for patients with advanced disease of aggressive histology [10,14,16,17]. A combination of short course chemotherapy and radiotherapy offers optimum cure rates with lower toxicity in these tumors [15]. Survival rates have improved with the addition of Rituximab to CHOP chemotherapy and further studies investigating the benefits of adding monoclonal antibodies to chemotherapy and radiotherapy are ongoing [18].

Radiotherapy which is considered the standard treatment for indolent orbital lymphomas, has also been found to be successful in aggressive NHL in some reports [5,13,14]. The doses recommended vary according to the site and histology of NHL, with no clear dose response yet evident [12-14]. In view of the fact that the prognosis of lacrimal sac lymphoma is good, radiation doses of 30 – 45 Gy may be recommended for these localized lesions with limited long term morbidity [13,14].

## Conclusion

The present result suggests that radial radiotherapy alone may be a treatment option with curative potential for primary localized NHL of the lacrimal sac and may be considered in patients who cannot tolerate appropriate chemotherapy.

## Competing interests

The author(s) declare that they have no competing interests.

## Authors' contributions

**RV, MKG **conception and design, acquisition of data, analysis and interpretation of data have been involved in drafting the manuscript, revising it critically for important intellectual content and have given final approval of the version to be published.
